# Supplementary material for: Identification and functional analysis of non-coding regulatory small RNA FenSr3 in Bacillus amyloliquefaciens LPB-18
Source: PeerJ. 2023 May 15;11:e15236. doi: 10.7717/peerj.15236 (PMC10194069; doi:10.7717/peerj.15236)
Supplement: Supplemental Information 4 [file peerj-11-15236-s004.zip › KO/CK-vs-T1_map/map05145.html]

KEGG PATHWAY: Toxoplasmosis - Reference pathway


|  |  |
| --- | --- |
| **Toxoplasmosis - Reference pathway** |  |

[
Pathway menu
| Organism menu
| Pathway entry
| Show description
| User data mapping
]

Toxoplasma gondii is an obligate intracellular parasite that is prevalent worldwide. The tachyzoite form acquired by oral ingestion downmodulates proinflammatory signaling pathways via various mechanisms. During early infection, nuclear translocation of NFkB is temporally blocked and p38 MAPK phosphorylation is prevented, suppressing IL-12 production. Another pathway for IL-12 induction occurs through CCR5 dependent pathway, but parasitic induction of an eicosanoid LXA4 contributes to the downregulation of IL-12. Direct activation of STAT3 by the parasite enhance anti-inflammatory function of IL-10 and TGF beta. T. gondii can cause lifelong chronic infection by establishing an anti-apoptotic environment through induction of bcl-2 or IAPs and by redirecting LDL-mediated cholesterol transport to scavenge nutrients from the host.

|  |  |  |
| --- | --- | --- |
| Reference pathway | 184% 150% 122% 100% 82% 67% 55% | 图片下载 |
